# Navigating the Complexities of Alzheimer’s Clinical Trials: The Perils of Small Molecule Studies and the Risk of Fraudulent Practices

**DOI:** 10.1002/alz.093551

**Published:** 2025-01-09

**Authors:** Patrick P O'Keefe, Suzanne B. Hendrix, Jeffery Zhang, Kent Hendrix, Lixia Wang, Samuel P. Dickson

**Affiliations:** ^1^ Pentara Corporation, Salt Lake City, UT USA; ^2^ Drome Burgundy, San Francisco, CA USA

## Abstract

**Background:**

Alzheimer’s disease (AD) presents unique challenges in clinical trials involving small molecules. Multifaceted issues plague such trials, emphasizing susceptibility to fraud from clinical sites and “professional patients”. The relative ease of simulating Alzheimer’s diagnosis, coupled with inadequate oversight by Contract Research Organizations (CROs), creates fertile ground for deceptive practices. Poor rater quality and the simplicity of falsifying data exacerbate concerns.

A core difficulty is the diagnostic ambiguity of AD, where symptomatic overlap with other cognitive disorders often leads to misdiagnosis or feigned conditions by professional patients. This issue is intensified in small molecule studies, which are easier to replicate or substitute than biologics, increasing the temptation and feasibility of fraudulent activities. Lax monitoring and control by CROs contribute to these vulnerabilities, allowing unnoticed data manipulation and deceitful conduct. It is particularly alarming that sites most prone to deceitful practices include predominantly underrepresented populations, thwarting inclusion efforts.

**Method:**

We critically analyze the inefficacy of regulatory audits in detecting and preventing fraud as clinical sites adeptly prepare regulatory documents to mask misconduct. We evaluate methods for detecting fraudulent practices based on the clinical data and clinical operations metrics.

**Result:**

The absence of medical expertise among auditors limits their ability to discern data integrity issues and the nuances of AD symptomatology. Traditional data management review and statistical analysis methods fail to identify these types of issues.

**Conclusion:**

We call for a reevaluation of current methodologies in AD clinical trials. We advocate for enhanced CRO oversight, improved rater training, and incorporation of medical expertise in audits. Targeted, blinded statistical data review can identify problematic sites. Addressing these challenges can safeguard AD research integrity, accelerate development of effective therapeutics, and protect and benefit vulnerable populations.

